# Circulating PUFAs and their associations with abdominal obesity and hyperglycaemia among vegetarians and non-vegetarians: Insights for personalised nutrition from a cross-sectional study

**DOI:** 10.1371/journal.pone.0337509

**Published:** 2026-01-07

**Authors:** Yuan Kei Ching, Yit Siew Chin, Mahenderan Appukutty, Yoke Mun Chan

**Affiliations:** 1 Department of Nutrition, Faculty of Medicine and Health Sciences, Universiti Putra Malaysia, Selangor, Malaysia; 2 Research Centre of Excellence, Nutrition and Non-Communicable Diseases, Universiti Putra Malaysia, Selangor, Malaysia; 3 Programme of Sports Science, Faculty of Sports Science and Recreation, Universiti Teknologi MARA, Shah Alam, Selangor, Malaysia; 4 Department of Dietetics, Faculty of Medicine and Health Sciences, Universiti Putra Malaysia, Selangor, Malaysia; Sarich Neuroscience Research Institute, AUSTRALIA

## Abstract

This study explores the associations between circulating polyunsaturated fatty acids (PUFAs), abdominal obesity and hyperglycaemia among vegetarians and non-vegetarians with distinct dietary PUFA intakes, as limited evidence exists on how circulating PUFA concentrations differ between these dietary groups and relate to metabolic risks. In this cross-sectional study, serum PUFA profiles and their associations with abdominal obesity and hyperglycaemia were examined**.** A total of 127 vegetarians and 132 non-vegetarians from Malaysia participated in the present study. Vegetarians had higher circulating concentrations of linoleic acid (LA), alpha-linolenic acid (ALA), and the *n-*6:*n-*3 PUFA ratio. They had lower concentrations of eicosapentaenoic acid (EPA), docosapentaenoic acid (DPA), and docosahexaenoic acid (DHA) than non-vegetarians. Among vegetarians, higher circulating arachidonic acid (ARA) concentrations were associated with an increased risk of hyperglycaemia (odds ratio [OR]: 1.06, 95% confidence interval [CI]: 1.00–1.11). In non-vegetarians, higher circulating ARA was associated with a higher risk of abdominal obesity (OR: 1.05, 95% CI: 1.00–1.09), while lower circulating DHA was associated with a reduced odds of abdominal obesity (OR: 0.95, 95% CI: 0.90–0.99). Conversely, a high circulating *n-*6:*n-*3 PUFA ratio was associated with a lower risk of hyperglycaemia (OR: 0.96, 95% CI: 0.93 - 1.00) among non-vegetarians. These findings highlight distinct metabolic responses to PUFA profiles between vegetarians and non-vegetarians, suggesting the need for tailored dietary strategies to address abdominal obesity and hyperglycaemia for both groups.

## Introduction

Diabesity is one of the significant public health concerns in the Southeast Asia region due to its rapidly increasing prevalence. It is characterised by the coexistence of obesity and hyperglycaemia, which are closely linked to the development of non-communicable diseases and fatty liver disease [1]. Among the various fat distributions in the human body, abdominal obesity contributes to the development of diabetes mellitus because excess abdominal fat can cause insulin resistance, inflammation and glucose metabolism dysregulation [2]. Both conditions are increasing rapidly worldwide and in Southeast Asia. Meta-analyses found that an estimated 41.5% of the world’s population or 2 in 5 adults has abdominal obesity [3]. In Southeast Asia, 30.0% to 50.0% of adults suffer from abdominal obesity. Alarmingly, Malaysia is the country that reports the highest cases of abdominal obesity (54.5%) compared to other countries in the same region [4]. Meanwhile, the International Diabetes Federation highlights that 537 million adults had diabetes mellitus in 2021, and their number is expected to rise to 783 million or 1 in 8 by 2045 [5]. In Southeast Asia, an estimated 90 million adults were diagnosed with diabetes mellitus in 2021, and this number is expected to rise to 69.0% in the next 20 years. In Malaysia, the National Health and Morbidity Survey found that 15.6% of Malaysians reported diabetes mellitus [4].

These metabolic trends coincide with the ongoing nutrition transition in Southeast Asia, marking a shift from traditional diets to Westernised diets, driven by urbanisation and globalisation [6]. In Southeast Asia, the ongoing nutrition transition has resulted in rising rates of abdominal obesity and hyperglycaemia. Alongside the nutrition transition, vegetarianism has grown in popularity over the past few decades. A significant increase in people practising vegetarian diets has been observed in various countries. For instance, the vegetarian diet is one of the most searched diets among the population in Malaysia, and an upward trend in vegetarian-related publications has been observed in the country over the past few years [7], highlighting the growing trend of vegetarianism among Malaysians. However, the benefits and drawbacks of a vegetarian diet compared to a non-vegetarian diet have always been a topic of debate due to the inconsistent findings reported in the literature. The nutritional adequacy of both vegetarian and non-vegetarian diets depends largely on balance of essential nutrients, whereby inadequate planning may lead to deficiencies in certain nutrients such as polyunsaturated fatty acids, vitamins, and minerals. These nutrients play critical role in maintaining metabolic health. Considering the disparities found across different regions, local studies are essential to better understand how dietary practices influence the development of abdominal obesity and hyperglycaemia within the community.

Circulating polyunsaturated fatty acids (PUFAs) also play roles in the distribution of body fat and regulation of blood glucose. There are two families of PUFAs, which are *n-*6 PUFAs, derived from linoleic acid (LA) and *n-*3 PUFAs, derived from α-linolenic acid (ALA). Both are essential PUFAs obtained from daily diet and further converted into long-chain polyunsaturated fatty acids (LC-PUFAs) such as eicosapentaenoic acid (EPA) docosapentaenoic acid (DPA) and docosahexaenoic acid (DHA) [8]. The dietary ratio of *n-*6:*n-*3 PUFAs is particularly important in controlling the endogenous synthesis of ARA, EPA, and DHA, with the recommended dietary intake ratio of *n-*6:*n-*3 PUFAs ranging from 3:1–5:1 [9]. However, the average dietary ratio of *n-*6:*n-*3 PUFAs in today’s diet has increased from 1:1–30:1 [9]. A higher proportion of dietary LA than dietary ALA may increase the endogenous production of ARA and decrease circulating EPA and DHA, has been linked to severe inflammation, especially in the presence of genetic predisposition [9]. These metabolic effects are consistent with population-based evidence. Epidemiological studies have reported that higher circulating n-6 PUFAs, particularly arachidonic acid (ARA), are associated with greater adiposity and risk of diabetes, whereas higher n-3 PUFAs (EPA and DHA) are linked to improved insulin sensitivity and lower body fat. Despite evidence linking PUFAs to metabolic health, no study has examined how circulating PUFA profiles relate to abdominal obesity and hyperglycaemia among vegetarians and non-vegetarians in Malaysia. Therefore, this study aimed to determine the circulating PUFA patterns and their associations with abdominal obesity and hyperglycaemia in these two dietary groups.

Meanwhile, circulating PUFAs reflect both dietary intake and endogenous metabolism. Despite vegetarians can still obtain n-3 PUFAs from plant-based sources such as nuts and seeds, these foods mainly provide ALA, and the endogenous conversion to EPA and DHA is limited. Vegetarians typically have lower circulating concentrations of EPA and DHA than non-vegetarians [10], which may put them at higher risk of abdominal obesity and hyperglycaemia. Conversely, non-vegetarians obtain EPA and DHA from fish and meat but may consume excessive LA and saturated fats from vegetable oils and animal products. PUFA metabolism also varies with genetic variation, particularly the fatty acid desaturase gene cluster, which influences the conversion of dietary ALA to circulating EPA and DHA and may have different effects on metabolic health depending on dietary habits. Overall, characterising PUFA metabolism across different dietary patterns can enhance understanding of metabolic risk factors and guide the development of targeted dietary interventions.

## Materials and methods

### Study design and respondents

The present study was a cross-sectional study conducted at nine randomly selected community centres in the Klang Valley (Kuala Lumpur and Selangor), Malaysia. These sites were chosen as study locations as Selangor is a region with the highest population density and Kuala Lumpur is the capital of Malaysia. The representatives of the selected community centres informed their members about the details of the present study through the leaflets prepared by the research team. Data collection was conducted from January 2022 to August 2022. All members who met the following inclusion criteria, namely Malaysians who were aged more than 18 years old, currently not pregnant or lactating, and not taking any fatty acids supplements and medications to control blood glucose, were invited to take part in the present study. Written informed consent was obtained from all members before their participation in the study.

The study protocol was approved by the Ethics Committee for Research Involving Human Subjects of Universiti Putra Malaysia (JKEUPM-2021–291) before the commencement of the data collection.

### Socio-demographic characteristics

Both vegetarians and non-vegetarians were asked to indicate their socio-demographic characteristics such as sex and age using a set of a self-administered questionnaire.

### Anthropometric measurements

Measurements of body weight and height were recorded as kilogram (kg) and centimetre (cm) by using a SECA213 portable stadiometer (SECA, Hamburg, Germany) and a TANITA Digital Weight Scale HD306 (TANITA Corporation, USA), respectively. The Lufkin tape (W606PM, Lufkin, USA) was used to measure the waist circumference of respondents in centimetres (cm) to the nearest 0.1 cm. Respondents were instructed to stand barefoot. The measurement was taken at the midpoint between the lower costal border and the iliac crest. Respondents were identified as having abdominal obesity if their waist circumference was 90.0 cm or more for males and 80.0 cm or more for females, based on Asian cut-offs [11].

### Dietary assessment

Trained researchers used a semi-quantitative food frequency questionnaire (FFQ) to record the dietary intake of vegetarians and non-vegetarians. The dietary intake of vegetarians was assessed using a semi-quantitative Food Frequency Questionnaire (FFQ) administered by trained researchers [19]. Vegetarians and non-vegetarians were provided with household measurements and food albums during the dietary intake interview. The FFQ consisted of 238 food items and was previously validated for use among Malaysian adults to assess habitual dietary intake. A food album and a set of household measurements were used during the dietary assessment to increase data accuracy. food items in the FFQ were classified into 16 groups, including whole grains. refined cereals and grains, legumes and their products, nuts and seeds, vegetables, fruits, sugars and syrups, Western fast foods, preserved and pickled products, textured vegetable protein (TVP) products, beverages, condiments, eggs, milk and milk products, oils and fats, and confectionaries and snacks. The classification of food groups was based on the FFQ used in the Malaysian Adult Nutrition Survey. PUFA intake (including linoleic acid, α-linolenic acid, and long-chain n-3 PUFAs) was estimated using the fatty acid composition data from the Malaysian Food Composition Database, complemented by the USDA database when local data were unavailable. The primary dietary sources of PUFAs among participants included vegetable oils, nuts, seeds, soy products, and fish-based dishes (for non-vegetarians). The selected nutrients (PUFAs, LA, ALA, EPA and DHA) were analysed using Nutritionist Pro Software Version 4.0.0 (First Data Bank, Axxya Systems, San Bruno, CA).

### Biochemical analyses

Respondents were instructed to fast for 8 hours prior to blood collection. A total of 2 ml of fasting blood was collected by registered phlebotomists for a fasting blood glucose test. The laboratory staff analyzed the blood glucose and lipid profile using an Olympus Au analyzer (AU640, Beckman Olympus, Tokyo, Japan). Respondents with fasting blood glucose concentration equal to or greater than 5.6 mmol/L were classified as having hyperglycaemia [11]. A total of 3 ml of the collected fasting blood specimen was used determine the circulating PUFAs composition. Serum was separated from the fasting blood specimen and stored at −80°C until further analysis and tested using the gas chromatography by trained laboratory staffs. Serum total lipids were extracted using the Folch method with chloroform: methanol (2:1, v/v), followed by transmethylation to form fatty acid methyl esters (FAMEs). FAMEs were analysed using a Shimadzu GC-2010 gas chromatograph (Shimadzu, Japan) equipped with a flame ionization detector (FID) and a cyanopropyl polysiloxane capillary column (SP-2560, 100 m × 0.25 mm i.d., 0.2 µm film thickness; Sigma-Aldrich) The concentration (mg/L) of each PUFA was calculated based on the peak area ratio of the target fatty acid to the internal standard, multiplied by the known concentration of the internal standard and adjusted for serum volume. The circulating PUFAs were recorded as mg/L for both vegetarians and non-vegetarians.

### Statistical analyses

Statistical analysis was conducted using IBM SPSS Statistics 26.0 (SPSS Inc., Chicao, IL, USA). All data were checked and only participants with complete information such social-demographic characteristics, anthropometric measurements and circulating PUFAs included in the analysis. Continuous variables that fall within skewness of ±2 were considered normally distributed [12] and were expressed as mean ± standard deviation (SD). Independent samples *t*-test was used to test the mean differences of continuous variables between vegetarians and non-vegetarians. On the other hand, categorical variables were presented as numbers (%) and tested using Pearson’s chi-square test. Multiple logistic regression was used to determine the associations of circulating PUFAs with abdominal obesity and hyperglycaemia. All variables with *p* < 0.25 in the simple logistic regression model proceeded with multiple logistic regression, which was adjusted for sex, age and ethnicity as covariates. Multicollinearity among the variables was checked and variables with VIF more than 10 were excluded. Linearity of the logit for continuous predictors was checked to ensure model validity. The odds ratios (ORs) and 95% confidence intervals (CIs) were determined. All variables with *p* < 0.05 were considered statistically significant.

## Results

Table 1 shows the socio-demographic characteristics, prevalence of abdominal obesity and hyperglycaemia as well as dietary PUFAs and circulating PUFAs among vegetarians and non-vegetarians. The present study involved a total of 259 respondents (127 vegetarians and 132 non-vegetarians) with more than half of the vegetarians (66.9%) and non-vegetarians (62.9%) being female. The mean ages of vegetarians and non-vegetarians were 41 ± 10 and 37 ± 12, respectively. For dietary PUFAs, vegetarians had higher concentrations of dietary *n-*6 PUFAs, LA and ALA than non-vegetarians (*p* < 0.05). While the non-vegetarians had higher concentrations of dietary EPA and DHA than vegetarians in our present study (*p* < 0.05). In the present study, a total of 191.00 ± 90.35 mg/L circulating PUFAs and 172.68 ± 92.18 mg/L circulating PUFAs were identified for vegetarians and non-vegetarians, respectively. The total circulating *n-*6 PUFAs, LA, ALA and ARA in vegetarians were significantly higher than in non-vegetarians (*p* < 0.05). On the other hand, non-vegetarians had significantly higher circulating concentrations of *n-3* PUFAs, EPA, DPA and DHA than vegetarians (*p* < 0.05). In terms of the circulating *n-*6:*n-*3 PUFA ratio, vegetarians had a significantly higher ratio of *n-*6:*n-*3 PUFAs than non-vegetarians in our study.

**Table 1:** General characteristics, dietary PUFAs and circulating PUFAs profiles of vegetarians and non-vegetarians.

Variables	Vegetarians (*n *= 127)	Non-vegetarians (*n* = 132)	*p* value
**Age (years)**	41 ± 10	37 ± 12	0.005*
**Sex**			0.495
Male	42 (33.1)	49 (37.1)	
Female	85 (66.9)	83 (62.9)	
**Ethnicity**			0.022*
Chinese	69 (54.3)	90 (68.2)	
Indian	58 (45.7)	42 (31.8)	
**Education level**			0.199
Primary level or lower	7 (5.5)	9 (6.8)	
Secondary level	69 (54.3)	84 (63.6)	
Tertiary level	51 (40.2)	39 (29.6)	
**Marital status**			0.308
Single	42 (33.0)	54 (40.9)	
Married	74 (58.3)	71 (53.8)	
Divorced/Widowed	11 (8.7)	7 (5.3)	
**Household income**			0.533
< RM 2300	34 (26.8)	44 (33.3)	
RM 2300–5599	43 (33.9)	56 (42.4)	
≥ RM 5600	50 (39.4)	32 (24.3)	
**Body weight (kg)**	62.81 ± 12.91	64.21 ± 14.84	0.418
**Height (cm)**	162.33 ± 8.71	161.85 ± 8,93	0.682
**BMI (kg/m**^**2**^)	23.86 ± 4.02	24.56 ± 4.98	0.241
**Abdominal obesity**			0.076
No	67 (52.8%)	84 (77.0)	
Yes	60 (47.2%)	48 (55.0)	
**Hyperglycaemia**			0.001*
No	109 (85.8)	91 (68.9)	
Yes	18 (14.2)	41 (31.1)	
**Dietary PUFAs intake**			
PUFAs (g/day)	14.27 ± 9.43	12.76 ± 6.77	0.140
*n-*6 PUFAs (g/day)	12.17 ± 6.77	10.27 ± 6.03	0.036*
LA	12.17 ± 8.24	10.27 ± 6.03	0.036*
*n-*3 PUFAs (g/day)	1.67 ± 1.60	1.65 ± 1.58	0.964
ALA (g/day)	1.64 ± 1.62	0.90 ± 0.56	<0.001*
EPA (g/day)	0.001 ± 0.001	0.200 ± 0.022	<0.001*
DHA (g/day)	0.02 ± 0.01	0.54 ± 0.15	<0.001*
*n-*6:*n-*3 PUFAs ratio	9.21 ± 4.98	8.46 ± 4.10	0.187
**Circulating PUFAs**			
PUFAs (mg/L)	191.00 ± 90.35	172.68 ± 92.18	0.108
*n-6* PUFAs (mg/L)	177.90 ± 85.14	151.26 ± 84.94	0.012*
LA (mg/L)	160.41 ± 79.57	137.78 ± 76.71	0.020*
ARA (mg/L)	15.66 ± 9.52	11.93 ± 11.60	0.005*
*n-3* PUFAs (mg/L)	13.10 ± 7.38	21.42 ± 13.05	<0.001*
ALA (mg/L)	1.99 ± 1.85	1.32 ± 0.88	<0.001*
EPA (mg/L)	0.99 ± 0.67	2.41 ± 2.26	<0.001*
DPA (mg/L)	3.17 ± 2.82	5.29 ± 5.01	<0.001*
DHA (mg/L)	6.94 ± 5.15	12.04 ± 9.98	<0.001*
*n-*6:*n-*3 PUFA ratio	15.83 ± 7.83	8.92 ± 5.94	<0.001*

Note: LA, linoleic acid; ALA, α-linolenic acid; ARA, arachidonic acid; EPA, eicosapentaenoic acid; DPA, docosapentaenoic acid; DHA, docosahexaenoic acid; PUFA, polyunsaturated fatty acid. Values are expressed in mean ± SD and n (%). Variables are tested by Independent samples *t*-test and Chi-square test. *Indicates a significant difference at *p* < 0.05.

Table 2 presents the circulating PUFA profiles in relation to abdominal obesity and hyperglycaemia among vegetarians and non-vegetarians. For abdominal obesity, no significant differences were found in all types of circulating PUFAs for vegetarians and non-vegetarians, respectively. Of those circulating PUFAs, the circulating *n-*6:*n-*3 PUFA ratio among vegetarians (*p = *0.077) as well as ARA (*p = *0.064) and circulating DHA (*p = *0.095) among non-vegetarians showed trends towards association with abdominal obesity. For hyperglycaemia, vegetarians with hyperglycaemia reported with higher circulating concentrations of total circulating PUFAs (*p = *0.045), circulating *n-*6 PUFAs (*p = *0.042) and circulating ARA (*p = *0.032) than vegetarians without hyperglycaemia. For non-vegetarians, non-vegetarians with hyperglycaemia had a lower concentration of *n-*3 PUFAs (*p = *0.024) than non-vegetarians without hyperglycaemia. These findings from bivariate analyses highlight that while circulating PUFAs may not be strongly associated with abdominal obesity, specific PUFAs, particularly total circulating PUFAs, ARA, and total *n-*3 PUFAs may influence hyperglycaemia in both vegetarians and non-vegetarians.

**Table 2:** Circulating PUFA profiles in relation to abdominal obesity and hyperglycaemia among vegetarians and non-vegetarians.

Variables	Abdominal obesity			Hyperglycaemia		
	No	Yes	*p* value	No	Yes	*p* value
**Vegetarians**						
Abdominal obesity, *n* (%)	67 (52.8%)	60 (47.2%)	–	–	–	–
Hyperglycaemia, *n* (%)	–	–	–	109 (85.8%)	18 (14.2%)	–
PUFAs (mg/L)	181.33 ± 70.45	201.81 ± 107.98	0.203	185.71 ± 93.01	223.06 ± 65.45	0.045*
*n-6* PUFAs (mg/L)	168.37 ± 67.15	188.55 ± 101.09	0.183	172.87 ± 87.69	208.35 ± 61.16	0.042*
LA (mg/L)	151.20 ± 60.51	170.71 ± 96.03	0.169	156.17 ± 82.11	186.15 ± 57.23	0.064
ARA (mg/L)	15.47 ± 9.57	15.88 ± 9.55	0.812	14.93 ± 9.18	20.11 ± 10.60	0.032*
*n-3* PUFAs (mg/L)	12.96 ± 5.52	13.26 ± 9.06	0.819	12.84 ± 7.19	14.70 ± 8.48	0.322
ALA (mg/L)	1.97 ± 1.09	2.01 ± 1.45	0.894	2.01 ± 1.97	1.90 ± 0.96	0.818
EPA (mg/L)	1.03 ± 0.64	0.95 ± 0.71	0.524	1.03 ± 0.67	0.77 ± 0.70	0.131
DPA (mg/L)	3.03 ± 2.21	3.39 ± 2.00	0.544	3.07 ± 2.80	3.79 ± 2.95	0.323
DHA (mg/L)	6.93 ± 3.99	6.96 ± 6.23	0.976	6.73 ± 4.61	8.25 ± 7.73	0.247
*n-*6:*n-*3 PUFA ratio	14.67 ± 6.82	17.12 ± 8.70	0.077	15.78 ± 8.12	16.14 ± 5.98	0.856
**Non-vegetarians**						
Abdominal obesity, *n* (%)	84 (63.6%)	48 (36.4%)	–	–	–	–
Hyperglycaemia, *n* (%)	–	–	–	91 (68.9%)	41 (31.1%)	–
PUFAs (mg/L)	169.34 ± 89.99	178.55 ± 96.59	0.583	176.31 ± 94.57	164.66 ± 87.27	0.504
*n-6* PUFAs (mg/L)	146.78 ± 81.14	159.11 ± 91.59	0.425	153.33 ± 86.16	146.67 ± 83.04	0.678
LA (mg/L)	134.78 ± 74.91	143.02 ± 80.30	0.555	140.53 ± 79.42	131.67 ± 70.88	0.541
ARA (mg/L)	10.53 ± 9.34	14.41 ± 14.53	0.064	11.33 ± 9.47	13.29 ± 15.37	0.371
*n-3* PUFAs (mg/L)	22.56 ± 14.63	19.44 ± 9.51	0.187	22.97 ± 13.87	17.99 ± 10.35	0.024*
ALA (mg/L)	1.36 ± 0.92	1.25 ± 0.81	0.489	1.31 ± 0.92	1.33 ± 0.82	0.920
EPA (mg/L)	2.51 ± 2.36	2.25 ± 2.09	0.533	2.57 ± 2.44	2.06 ± 1.76	0.229
DPA (mg/L)	5.31 ± 5.03	5.27 ± 5.03	0.970	5.78 ± 5.22	4.20 ± 4.38	0.074
DHA (mg/L)	13.39 ± 11.08	10.67 ± 7.47	0.095	13.30 ± 10.55	10.40 ± 8.33	0.092
*n-*6:*n-*3 PUFAratio	8.60 ± 5.92	9.48 ± 6.01	0.416	8.32 ± 5.49	10.24 ± 6.73	0.086

Note: LA, linoleic acid; ALA, α-linolenic acid; ARA, arachidonic acid; EPA, eicosapentaenoic acid; DPA, docosapentaenoic acid; DHA, docosahexaenoic acid; PUFA, polyunsaturated fatty acid.. Values are expressed in mean ± SD. Variables are tested by Independent samples *t*-test. *Indicates a significant difference at *p* < 0.05.

Table 3 displays the odds ratios of circulating PUFAs with abdominal obesity and hyperglycaemia for vegetarians using multiple logistic regression. The findings indicate that none of them significantly contribute to abdominal obesity, except for the circulating *n-*6:*n-*3 PUFA ratio. The circulating *n-*6:*n-*3 PUFA ratio was close to the significance level (OR: 1.04, 95% CI: 0.99–1.09) (*p = *0.083), which suggest a potential trend in which a higher *n-*6:*n-*3 ratio may contribute to an increased likelihood of abdominal obesity. For hyperglycaemia, higher circulating ARA significantly contributed to an increased odds of hyperglycaemia (OR: 1.06, 95% CI: 1.00–1.11) (*p = *0.037). This finding suggests that excessive circulating ARA, a precursor of pro-inflammatory eicosanoids may contribute to insulin resistance and impaired glucose metabolism.

**Table 3:** Associations of dietary and circulating PUFAs with abdominal obesity and hyperglycaemia for vegetarians (*n *= 127).

Variables	Univariate logistic regression	*p* value	Multiple logistic regression	*p* value
	Crude OR (95% CI)		Adjusted OR (95% CI)	
**Abdominal obesity**				
PUFAs (mg/L)	1.00 (0.99–1.01)	0.219	–	–
*n-6* PUFAs (mg/L)	1.00 (0.99–1.01)	0.199	–	–
LA (mg/L)	1.00 (0.99–1.01)	0.187	–	–
ARA (mg/L)	1.01 (0.97–1.04)	0.810	–	–
*n-3* PUFAs (mg/L)	1.01 (0.96–1.05)	0.818	–	–
ALA (mg/L)	1.01 (0.84–1.22)	0.893	–	–
EPA (mg/L)	0.84 (0.50–1.42)	0.521	–	–
DPA (mg/L)	1.04 (0.92–1.18)	0.543	–	–
DHA (mg/L)	1.00 (0.94–1.07)	0.976	–	–
*n-*6:*n-*3 PUFA ratio	1.04 (0.99–1.09)	0.083	1.04 (0.99–1.09)	0.083
**Hyperglycaemia**				
PUFAs (mg/L)	1.00 (0.99–1.01)	0.142	–	–
*n-6* PUFAs (mg/L)	1.00 (1.00–1.01)	0.138	–	–
LA (mg/L)	1.00 (0.99–1.01)	0.175	–	–
ARA (mg/L)	1.06 (1.00–1.11)	0.037	1.06 (1.00–1.11)	0.037*
*n-3* PUFAs (mg/L)	1.03 (0.97–1.09)	0.331	–	–
ALA (mg/L)	0.96 (0.70 −1.32)	0.817	–	–
EPA (mg/L)	0.51 (0.22–1.23)	0.135	–	–
DPA (mg/L)	1.08 (0.93–1.25)	0.331	–	–
DHA (mg/L)	1.05 (0.97–1.14)	0.256	–	–
*n-*6:*n-*3 PUFA ratio	1.01 (0.95 −1.07)	0.855	–	–

Note: LA, linoleic acid; ALA, α-linolenic acid; ARA, arachidonic acid; EPA, eicosapentaenoic acid; DPA, docosapentaenoic acid; DHA, docosahexaenoic acid; PUFA, polyunsaturated fatty acid. Variables with *p* < 0.25 in simple logistic regression were entered into the multiple logistic regression. Backward likelihood ratio method was applied to determine the optimise factors contributing towards abdominal obesity and forward likelihood ratio towards hyperglycaemia. For abdominal obesity, classification table (overall percentage: 57.5%), Cox & Snell *R*^*2*^ (0.025) and Nagelkerke *R*^*2*^ (0.033). For hyperglycaemia, classification table (overall percentage: 85.8%), Cox & Snell *R*^*2*^ (0.034) and Nagelkerke *R*^*2*^ (0.061). * Indicates a significant difference at *p* < 0.05.

Table 4 presents the odd ratios of circulating PUFAs contributing to abdominal obesity and hyperglycaemia for non-vegetarians using multiple logistic regression. Higher circulating ARA significantly contributed to abdominal obesity (OR: 1.05, 95% CI: 1.00–1.09) (*p = *0.017). In contrast, circulating DHA showed a protective effect (OR: 0.95, 95% CI: 0.90–0.99) (*p = *0.028), suggesting that higher circulating DHA concentration might reduce the risk of abdominal obesity. Regarding hyperglycaemia, a higher circulating *n-*6:*n-*3 PUFA ratio contributed to decreased odds of hyperglycaemia (OR:0.96, 95% CI: 0.93–1.00) (*p = *0.047).

**Table 4:** Associations of circulating PUFAs with abdominal obesity and hyperglycaemia for non-vegetarians (*n *= 132).

Variables	Univariate logistic regression	*p* value	Multiple logistic regression	*p* value
	Crude OR (95% CI)		Adjusted OR (95% CI)	
**Abdominal obesity**				
PUFAs (mg/L)	1.00 (0.99–1.01)	0.580	–	–
*n-6* PUFAs (mg/L)	1.00 (0.98–1.01)	0.422	–	–
LA (mg/L)	1.00 (0.99–1.01)	0.552	–	–
ARA (mg/L)	1.03 (0.99–1.06)	0.074	1.05 (1.00–1.09)	0.017*
*n-3* PUFAs (mg/L)	0.98 (0.95–1.01)	0.189	–	–
ALA (mg/L)	0.87 (0.58–1.30)	0.486	–	–
EPA (mg/L)	0.95 (0.81–1.12)	0.531	–	–
DPA (mg/L)	0.99 (0.93–1.07)	0.970	–	–
DHA (mg/L)	0.97 (0.93–1.01)	0.136	0.95 (0.90–0.99)	0.028*
*n-*6:*n-*3 PUFA ratio	1.03 (0.96–1.09)	0.415	–	–
**Hyperglycaemi**a				
PUFAs (mg/L)	0.99 (0.99–1.00)	0.501	–	–
*n-6* PUFAs (mg/L)	0.99 (0.99–1.00)	0.676	–	–
LA (mg/L)	0.99 (0.99–1.00)	0.538	–	–
ARA (mg/L)	1.01 (0.98–1.05)	0.372	–	–
*n-3* PUFAs (mg/L)	0.97 (0.93–1.00)	0.047	–	–
ALA (mg/L)	1.02 (0.67–1.55)	0.919	–	–
EPA (mg/L)	0.89 (0.75–1.07)	0.231	–	–
DPA (mg/L)	0.93 (0.86–1.01)	0.096	–	–
DHA (mg/L)	0.97 (0.9–1.01)	0.126	–	–
*n-*6:*n-*3 PUFA ratio	1.05 (0.99–1.12)	0.093	0.96 (0.93–1.00)	0.047*

Note: LA, linoleic acid; ALA, α-linolenic acid; ARA, arachidonic acid; EPA, eicosapentaenoic acid; DPA, docosapentaenoic acid; DHA, docosahexaenoic acid; PUFA, polyunsaturated fatty acid. Variables with *p* < 0.25 in simple logistic regression were entered into the multiple logistic regression. Backward likelihood ratio method was applied to determine the optimise factors contributing towards abdominal obesity and forward likelihood ratio towards hyperglycaemia. For abdominal obesity, classification table (overall percentage: 60.6%), Cox & Snell *R*^*2*^ (0.067) and Nagelkerke *R*^*2*^ (0.092). For hyperglycaemia, classification table (overall percentage: 68.9%), Cox & Snell *R*^*2*^ (0.034) and Nagelkerke *R*^*2*^ (0.048). * Indicates a significant difference at *p* < 0.05.

## Discussion

In the present study, both vegetarians and non-vegetarians face a similar prevalence of abdominal obesity, indicating that factors beyond diet, such as genetic predisposition and lifestyle may be associated with fat distributions. These findings similar with previous inconsistent evidence [13] and highlight the need for future research to conduct a comprenensive approach that considering the roles of various factors. In addition, considerable variation in food choices, portion sizes and nutrient quality within both vegetarian and non-vegetarian diets may affect obesity and metabolic outcomes, indicating that dietary classification alone may not fully capture the complex dietary influences on abdominal obesity and hyperglycaemia. As for hyperglycaemia, vegetarians in the present study had lower prevalence of hyperglycaemia than non-vegetarians, consistent with evidence that plant-based diets rich in fibre improve glycaemic control [14]. Increased fibre intake has been associated with better β-cell function, insulin sensitivity and postprandial glycaemic control [14]. These benefits are largely mediated by shifts in gut microbiota composition and activity, which can be directed towards two primary metabolic pathways: saccharolytic or proteolytic fermentation [15]. A high-fibre diet promotes saccharolytic fermentation, enriching for bacteria that produce the beneficial short-chain fatty acids (SCFAs) acetate, propionate, and butyrate. These SCFAs directly correlate with metabolic benefits, including elevated glucagon-like peptide-1, a reduction in HbA1c, and improved blood-glucose regulation [16].Conversely, a lack of dietary fibre promotes proteolytic fermentation, where bacteria metabolize protein into harmful post-biotics such as ammonia and phenols linked to inflammation and insulin resistance [17]. Excessive consumption of red meat has been associated with C-reactive protein (CRP) concentration, insulin resistance, oxidative stress, and inflammation, which may affect glucose metabolism and blood inflammation intensity [18]. These complications increase the risk of impaired glucose homeostasis, which may explain the higher incidence of hyperglycaemia among non-vegetarians than vegetarians.

Circulating PUFAs, particularly the reduced long-chain omega-3 concentration observed in vegetarians may associate with inflammation, lipid metabolism and visceral adiposity. The present serves the first local comparison of circulating PUFAs between vegetarians and non-vegetarians in Malaysia. Our observations of higher circulating LA in vegetarians are consistent with a recent study that compared the circulating PUFAs between vegetarians and non-vegetarians in Germany [19]. Measuring circulating PUFA concentrations provides a more objective and reliable indicator of long-term dietary patterns compared to estimates derived from self-reported dietary intake, as it reflects actual absorption and avoids the limitations of recall bias. Prio research has observed correlation between circulating LA concentrations and dietary LA intake vegetarians [19]. Thus, the elevated circulating LA in vegetarians than non-vegetarians may be explained by the higher dietary intake of LA in vegetarians compared to non-vegetarians.

Our findings show that vegetarians had a higher circulating concentrations of ALA but lower EPA and DHA compared to than non-vegetarians, which consistent with previous research The elevated ALA reflects greater dietary intake of ALA, whereas the lower EPA and DHA may result from limited direct sources such as fatty acid, restricted endogenous conversion of ALA influenced by high LA intake, genetic variation in desaturase activity, or micronutrient deficiencies affecting PUFA metabolism. The low circulating concentrations of EPA and DHA could be the low conversion rate of ALA to EPA and DHA along the fatty acids metabolism pathway encoded by the fatty acid desaturase gene [20]. Moreover, the higher circulating LA in vegetarians could competitively inhibit conversion of ALA to EPA and DHA, further explaining these differences. It is very challenging to achieve the recommended n-6:n-3 PUFA ratio of 3:1–5:1 [9] as most of plant-based foods such as vegetable oil, pistachio, almonds and walnuts contain more of LA than ALA. Only a few sources such as flaxseed and hemp seed provide amount of ALA [21]. In the present study, both vegetarians and non-vegetarians exceeded the recommended *n-*6:*n-*3 PUFA ratio, whereby vegetarians showing a particularly high n-6:n-3 ratio [10]. The imbalance is probably due to elevated circulating *n-6* PUFA(LA and ARA) and lower *n-*3 PUFAs (EPA, DPA and DHA). A higher *n-*6:*n-*3 PUFA ratio may increase inflammation, especially under genetic predisposition [9]. Our study indicates that most circulating PUFAs are not associated with abdominal obesity, except the *n-*6:*n-*3 PUFA ratio, which approached significance among vegetarians. High circulating *n-*6 PUFAs has been associated with inflammation, fat storage and oxidative stress [22]. Vegetarians may benefit from increasing *n-*3 PUFAs intake through flaxseed, chia seeds and nuts to maintain a favourable *n-*6:*n-*3 ratio. In non-vegetarians, elevated circulating ARA was associated with abdominal obesity. Elevated circulating ARA exacerbates obesity through disruption in the gut-hypothalamus-adipose-liver axis, promoting proinflammatory bacteria and reduce the protective component such as butyrate [23]. Since vegetable oils such as soybean, corn and sunflower oil are often high in LA [24], limiting the processed foods manufactured by these oils might help to reduce abdominal fat accumulation. Conversely, circulating DHA demonstrated protective effects, which may be due to its ability to reduce adipocytes through anti-inflammatory and lipid-modulating effects [25]. Since humans can only improve circulating PUFAs through dietary changes, they should pay attention to their dietary PUFAs intake. Vegetarians may benefit from microalgae consumption to increase EPA and DHA, whereas non-vegetarians can obtain these fatty acids from fatty fish [8].

As for hyperglycaemia, the significant association of circulating ARA and hyperglycaemia among vegetarians suggest that excessive circulating ARA is associated with metabolic risk through inflammatory pathway. Circulating ARA acts as the precursor for pro-inflammatory eicosanoids such as prostaglandins and leukotrienes, which trigger oxidative stress and impair glucose regulation [26]. One of the notable concern is that the current circulating *n-*6:*n-*3 PUFA ratio is 9:1 exceeds the recommended ratio, which may contribute to chronic inflammation and increased insulin resistance among vegetarians [27]. An earlier report had found that the circulating ratio of *n-*6:*n-*3 PUFAs was higher in individuals with hyperglycaemia [28]. On the other hand, a systematic review mentions that increased *n*-6 circulating PUFAs did not cause any detrimental effects on glucose metabolism and diabetes mellitus [29]. The role of circulating n-6 PUFAs in hyperglycaemia appears complex. While univariate analysis suggested a higher n-6:n-3 ratio increased risk, multivariate analysis indicated a protective effect when considering all PUFAs simultaneously. This discrepancy may reflect interactions among different PUFAs, individual metabolic differences, or genetic variations in desaturase enzymes, highlighting the need for caution when interpreting n-6 PUFA effects in cross-sectional studies. In particular, genetic variation in the fatty acid desaturase (FADS) gene cluster, may alter the effect of circulating PUFAs on glucose homeostasis, potentially explaining discrepancies in the association with hyperglycaemia. While excessive *n-*6 PUFAs can promote inflammation, some sources of n-6 PUFAs such as nuts and seeds reducing hyperglycaemia [30]. In our study, a higher circulating circulating *n-*6:*n-*3 PUFA ratio was associated with a lower risk of hyperglycaemia. This association was determined after accounting the presence for multiple PUFAs in the multiple logistic regression model. This indicating that the overall balance of PUFAs, rather than any single type of PUFA plays more important role in regulating the glucose concentration. The current findings suggest that dietary strategies could be planned for individual PUFA profiles to support better metabolic health. For example, vegetarians may benefit from increasing n-3 PUFA intake since higher circulating *n-*6 PUFAs particularly circulating ARA, are positively associated with hyperglycemia and a high circulating *n-*6:*n-*3 PUFA ratio is identified as a potential risk factor for abdominal obesity. Conversely, non-vegetarians may consider moderating ARA intake and ensuring adequate DHA intake to lower inflammation and improve metabolic outcomes. These dietary implementations should be taken with careful consideration as overall diet quality, food sources, supplement use, genetic variants and lifestyle factors may also link to hyperglycemia and abdominal obesity. These findings indicate that individual PUFA profiles may influence metabolic outcomes. While causal relationships cannot be established in this cross-sectional study, the results suggest that future research could explore whether personalised dietary strategies targeting PUFA balance might improve metabolic health. In conjunction with these, genetic screening for fatty acid desaturase genes that regulate PUFAs metabolism, coupled with PUFAs-focused dietary counselling in healthcare settings, is required to facilitate the implementation of personalized nutrition. In the long term, Malaysia could surpass the use of one-size-fits-all recommendations and implement more effective dietary strategies addressing individuals’ specific needs to tackle abdominal obesity and hyperglycemia through personalised nutrition.

The present study provides a comprehensive overview of circulating PUFAs composition and their associations with abdominal obesity and hyperglycaemia among vegetarians and non-vegetarians, which is rarely explored in Malaysia. Nonetheless, the findings are subject to some limitations. First, its cross-sectional design captures associations between circulating PUFAs and abdominal obesity and hyperglycaemia at a single point in time, preventing the establishment of causal relationships. Longitudinal or intervention studies are needed to affirm the observed findings. Next, the relatively sample size may limit the statistical power to detect smaller associations. Furthermore, the results are derived from selected Malaysian populations, which suggests that the conclusions drawn may not be generalized to vegetarians and non-vegetarians in other regions, which may lead to selection bias.Future studies would benefit by including vegetarians and non-vegetarians from different areas.

## Conclusion

The present study found differentiating circulating PUFA profiles between vegetarians and non-vegetarians. Vegetarians exhibiting lower concentrations of anti-inflammatory PUFAs (EPA, DPA and DHA) and higher concentration of pro-inflammatory circulating ARA, which were differentially associated with abdominal obesity and hyperglycaemia. These observations suggest that variations in dietary patterns and fatty acid composition may contribute to metabolic differences between groups. Although the current findings showed the importance of maintaining balanced *n-*6*:n-*3 PUFA ratio, these should be interpreted with caution. Its cross-sectional design as well as the limited sample size restrict causal inference and generalisability. Future longitudinal and intervention studies can consider using a larger and more diverse populations to further determine how genetic and lifestyle factors contribute to abdominal obesity and hyperglycaemia. Despite this, the present study provides preliminary findings that may inform future research and recommendation on refining dietary recommendations in order to tailor dietary strategies in addressing abdominal obesity and hyperglycaemia across for both groups.
